# Oral Health and Caries Prevention: How Tongue Hygiene Helps Maintain Balance of Microbiota and Overall Health in Pediatric Patients

**DOI:** 10.3390/children11070816

**Published:** 2024-07-03

**Authors:** Giovanna Mosaico, Mara Pinna, Roberta Grassi, Germano Orrù, Andrea Scribante, Carolina Maiorani, Cinzia Casu, Gianna Maria Nardi, Andrea Butera

**Affiliations:** 1 Independent Researcher, 72012 Carovigno, Italy; 2 Department of Surgical Sciences, Oral Biotechnology Laboratory (OBL), University of Cagliari, 09121 Cagliari, Italy; m.pinna137@studenti.unica.it (M.P.); orru@unica.it (G.O.); ginzia.85@hotmail.it (C.C.); 3 Department of Oral Surgery, Tor Vergata University, 00100 Rome, Italy; roberta.grassi@students.uniroma2.eu; 4 Unit of Orthodontics and Pediatric Dentistry, Section of Dentistry, Department of Clinical, Surgical, Diagnostic and Pediatric Sciences, University of Pavia, 27100 Pavia, Italy; andrea.scribante@unipv.it; 5 Unit of Dental Hygiene, Section of Dentistry, Department of Clinical, Surgical, Diagnostic and Pediatric Sciences, University of Pavia, 27100 Pavia, Italy; carolinamaiorani@outlook.it (C.M.); andrea.butera@unipv.it (A.B.); 6 Department of Odontostomatological and Maxillofacial Sciences, Sapienza University of Rome, 00161 Rome, Italy; giannamaria.nardi@uniroma1.it

**Keywords:** caries prevention, pediatric patient, therapeutic savings, tongue cleaning, tongue microbiota, systemic diseases

## Abstract

Background/Objectives: The tongue harbors about two-thirds of the microorganisms present in the mouth; the stable bacterial population consists mainly of aerobic and facultative anaerobic streptococci. These bacterial colonies, found more frequently on the tongue than on the outside of the hard part of the dental enamel in children younger than 18 months, suggest that the tongue is a potential bacterial reservoir. The aim of this review is to examine the scientific literature to clarify whether the mechanical removal of bacterial biofilm on the tongue can have a positive effect on caries prevention, with the reduction in colony-forming unit (CFU) of salivary streptococcus and the whole-mouth plaque index (FMPS). Methods: An open literature search was conducted by using PubMed (MEDLINE), Cochrane Library and Google Scholar. The most studied age range was 9 to 12 years, with groups of children with no caries and groups with a minimum number of two teeth that were reconstructed, decayed and/or missing (DMFS/dmfs > 2) who experienced different tongue hygiene methods for the first time. Results: Four randomized trials met the search criteria and were included in this review. Conclusions: The results obtained suggest that specific tongue hygiene protocols, combined with a healthy diet and lifestyle, could be considered the gold standard to enable more effective primary prevention and improve the health of pediatric patients. This review improves the understanding of the impact of tongue hygiene in controlling the bacteria responsible for the onset of carious disease and its systemic correlates; however, further research with more data is needed to further confirm the findings of this research.

## 1. Introduction

Differentiated microbiotic habitats originate in the oral cavity at the level of the hard tissues of the teeth and in the soft tissues of the gingival sulcus, the back of the tongue, the lips, the cheeks and the palate [1]. The microhabitats inside the pediatric oral cavity differ significantly in the absolute abundance of bacteria; furthermore, during night sleep, the oral microbiota are particularly dynamic, and this phenomenon makes the oral cavity a fertile ground for bacterial proliferation, which is also encouraged by poor oral hygiene (OH) and the reduction in saliva flow during a long night’s rest [2]. The hard tissues represented by the teeth allow for the colonization of the aggregated bacterial biofilm, while the tongue and the soft tissues (mucous membranes) flake easily and constantly, allowing the bacterial biofilm to colonize other areas of the oral cavity more easily [3].

### 1.1. Etiology of Caries

Dental caries is an infectious, degenerative and destructive disease affecting the hard substances of the tooth (the enamel and dentine); if not treated, they progress in depth up to the dental pulp. It has a multifactorial etiology: bacterial plaque plays a crucial role in its pathogenesis, as does a diet rich in sugars and reduced defense capacity of the host against pathogenic microorganisms [4,5,6]. Other determining factors in the onset and progression of the carious process are the buffer capacity of saliva to neutralize the oral acid pH and the “time” factor, i.e., the synergistic action of food sugars and acidogenic bacteria—in particular, *Streptococcus mutans*, *Streptococcus sobrinus* and *Lactobacillus*. Socio-economic factors are also considered fundamental in the onset and progression of dental caries [7,8,9,10,11,12]. Caries is a disease of various etiologies; during its different phases, pH, diet and acidogenic bacteria are decisive in the demineralization of the enamel, while other bacteria of proteolytic origin—in addition to acidogenic bacteria—are involved in progression in the dentin. Thus, carious disease has a polymicrobial etiology [13,14,15]. The bacterial species associated with the onset and progression of caries include—in addition to *S. mutans—Actinomyces* and *Bifidobacterium* spp. [16]. Groups of bacteria such as *Streptococcus salivarius*, *S. sobrinus*, *Streptococcus parasanguinis*, *Scardovia wiggsiae*, *Slackia exigua*, *Lactobacillus salivarius*, *Parascardovia denticolens Porphyromonas*, *Actinomyces* and *Veillonella* have been detected in the oral microbiota of children with deep caries [17,18,19,20]. The bacterial species associated with the initiation and progression of caries (including *Streptococcus*, *Actinomyces* and *Lactobacillus* species) are microorganisms of saccharolytic origin, capable of metabolizing dietary carbohydrates to produce the lactic acid necessary to initiate the demineralization of the enamel [21]. Through sugars, lactate dehydrogenase is activated, with predominant production of lactate. Pyruvate kinase increases glycolysis and lactate production [22]. *Veillonella* species use lactate as the fundamental carbon and energy precursor molecule, switching it to pyruvate and succinate by means of enzymatic reactions. An overabundance of dietary carbohydrates increases the percentage of intracellular polysaccharides, which become reserves when the intake of extracellular sugars is reduced (e.g., between meals) [21]. Early childhood caries (ECC) is a carious disease that begins before the age of six and sometimes even before the age of two. It is a multifactorial disease attributable, in most cases, to uncontrolled intake of dietary sugars, including honey and sweet drinks in general. ECC has prevalence rates ranging from 12 to 27% among children aged 2 to 3 years [23,24,25] and from 27 to 48% among children aged 4 to 6 years. The impact of ECC in the United States is 3 to 28% [26,27,28]. In the oral microbiota of affected children younger than 36 months, *S. mutans* was found to be largely implicated in the most important form of ECC. Other responsible pathogens in severe ECC include *Scardovia wiggsiae* and the Bifidobacteriaceae species of *Porphyromonas* and *Actinomyces* bacteria [27,29,30]. *Candida albicans* plays a major role in ECC; in fact, it is frequently found in abundance in children with worrisome forms of ECC compared with children without caries [31].

### 1.2. Effects and Systemic Correlations of Dental Caries

Dental caries can have a negative impact on the health and quality of the young patient due to the pain, which causes a reduction in the appetite with consequent weight loss due to difficulties in chewing food and hypersensitivity to cold, heat and thermal and chemical stimuli, as well as acid in sugary foods and drinks. Furthermore, pain can compromise nighttime sleep, with negative consequences for academic performance [32,33]. Untreated dental caries can be the consequence of pathologies even far from the oral cavity in children with special needs who suffer from chronic conditions such as seizure disorders. These children may be more susceptible to pneumonia, urinary tract infections, fever, generalized infections, and endocarditis in pediatric patients with congenital heart valve defects [34]. Koerdt et al. found that *S. mutans* is the main pathogen present in the saliva of children with coronary heart disease [35]. Pulpitis and recurrent dental abscesses can slow down a child’s body growth due to chronic systemic inflammation that affects metabolic pathways [36]. The mechanism of action of this phenomenon could be attributed to proinflammatory cytokines such as interleukin-1 (IL-1), which can inhibit erythropoiesis by reducing the production of red blood cells in the bone marrow, resulting in anemia and chronic hematological disease [37].

### 1.3. Dental Bacterial Plaque

Microbial species, in particular bacteria, tend to organize themselves into biofilms to increase resistance to antibacterial agents, especially antibiotics, and ensure greater survival. Dental bacterial plaque is a biofilm formed by a polymicrobial aggregate that adheres to and colonizes the smooth surface of teeth, root surfaces and dental implants. It is incorporated into a polysaccharide matrix formed by mucins and antibodies, which contribute to forming the acquired film [38,39]. The acquired film also acts as a protective matrix of the enamel against acid and mechanical insults and develops on the surface of the teeth immediately after thorough home OH or professional deplaquing. Planktonic cells adhere to the acquired pellicle through adhesins present in the peripheral cells, which intercept the matrix proteins through indeterminate physicochemical connections [40].

### 1.4. Composition of Oral Bacterial Plaque

The oral biofilm is able to alter the balance of the host microbiota, initiate oral dysbiosis and promote the onset of caries and periodontal diseases with reflections on systemic health [41,42].

Biofilm is considered a complex and effectively organized bacterial system responsible for the two most widespread oral diseases: tooth decay and gingival-periodontal diseases. The different oral environments presuppose a substantial difference in the bacterial species involved in biofilm formation; therefore, the supragingival bacterial population differs from the subgingival one [43]. The primordial colonizing bacteria of the oral cavity area biofilm are streptococci (yellow complex), followed by *Actinomyces* species (green, blue and purple complexes) [44]. *Fusobacterium* species (orange complex) play a central role that essentially allows for biofilm maturation by binding together the Gram-positive colonizing bacteria (cocci) and the Gram-negative bacteria of the red complex (*Porphyromonas*). Thus, a complex range of bacterial species that are related to each other are efficiently, stably and firmly aggregated [45,46]. *Actinomyces* sp., *Streptococcus* sp., *Lactobacillus* sp., and *Candida* spp. constitute the most superficial substance of plaque. With plaque progression, through bacterial annexation, the transformation from a supragingival plaque of Gram-positive saccharolytic origin to a Gram-negative proteolytic plaque is determined by the accessibility of nutrients directly from saliva. Dissolved food nutrients in saliva represent the greater nourishment of supragingival plaque. In subgingival plaque, especially in deep pockets, the penetration of nutrients through saliva is limited; therefore, the major source of nourishment comes from tissues such as the periodontal and blood tissues. According to a study by Thurnheer et al., in subgingival plaque, *Actinomyces* sp. could survive and contribute to the formation of the dominant subgingival biofilm together with *Fusobacteria* and *Tannerella* sp. while *P. gingivalis*, *P. intermedia*, *P. endodontalis* and *P. micra* could form transient microcolonies of the biofilm [43].

### 1.5. Bacterial Biofilm on the Tongue

The oral district is populated by more than 700 species of microorganisms, including fungi, viruses and protozoa, where 58% have an official name, 16% are unnamed but have been cultivated and 26% are known only as uncultivated phylotypes (https://www.homd.org/, accessed on 16 June 2024) [47]. The tongue has the highest bacterial load of all oral tissues, hosting about two-thirds of the bacteria present in the oral cavity. These microorganisms are embedded in a thin mucus layer made up of complex extracellular polymeric substances [48,49]. In numerical terms, over 100 bacteria can adhere to every unique coating cell on the surface of the tongue’s mantle, while at other sites of the mouth, only about 25 bacteria can colonize a single epithelial cell. The stable bacterial population of the tongue is mainly made up of aerobic and facultative anaerobic streptococci (*S. salivarius*, *S. mitis* and *S. sanguis*); furthermore, *Veillonella* (coconut) may reside in the crypts of the papillae [50]. In the warm, humid environment of the tongue, there is a continuous migration of Gram-positive and Gram-negative bacteria, dead and disrupted cells and oral debris trapped between the folds and papillae, especially at the back. This physiological phenomenon is due to the dynamism of the biofilm present on the tongue, which is not organized and aggregated as on the tooth surfaces and the gingival edge, which develops a fairly independent microecosystem that pours easily into the saliva [51] and which could contribute significantly and exponentially to increasing the accumulation of bacterial plaque on the teeth [52,53,54,55]. In the microbiota of the dorsum of the tongue in children aged between 6 and 36 months, Tanner et al. detected, in the biofilm of the tongue coat of 171 children, *S. mutans* (the highest bacterial concentration of 70%), *S. sobrinus* (72%), *Porphyromonas gingivalis* (23%), *Bacteroides forsythus* (11%) and *Aggregatibacter actinomycetemcomitans* (30%). Bacterial species associated with caries included *S. mutans* in children aged between 18 and 36 months and *Actinomyces israelii* in children younger than 18 months; the latter species could be associated with an increase in bacterial plaque in children with caries. These bacterial colonies, found more frequently on the tongue than on the outside of the hard part of the tooth in children younger than 18 months, suggest that the tongue is a potential bacterial reservoir [56]. As a result, lowering the bacterial load on the tongue coat can decrease plaque buildup on the teeth by reducing the colony-forming unit (CFU) value of carious bacteria in saliva, helping to more effectively prevent tooth decay, gum/periodontal disease and related systemic diseases [57,58]. Proper oral hygiene, including tongue cleaning, is essential in the management and care of adult and pediatric patients in intensive care units (ICUs) to reduce the incidence of nosocomial infections that occur due to changes in oral microflora, typically within 48 h of presentation to the ICU [59].

## 2. Purpose of Work

The aim of this review is to examine the scientific literature to clarify whether the mechanical removal of bacterial biofilm from the posterior tongue can have a positive effect on caries prevention and on the reduction in salivary streptococcal CFU and whole-mouth plaque index (FMPS), in order to identify a preventive protocol validated in the literature, allow for more effective prevention of caries and improve the oral and systemic health of pediatric patients.

### 2.1. Materials and Methods

In order to provide a new guideline to healthcare professionals for the prevention of carious disease in pediatric patients, research was carried out on the inclusion of tongue cleaning in OH. We examined the English scientific literature, including literature reviews, case–control studies, cross-sectional studies and clinical studies, that investigated different tongue hygiene techniques in children.

**Figure 1:**
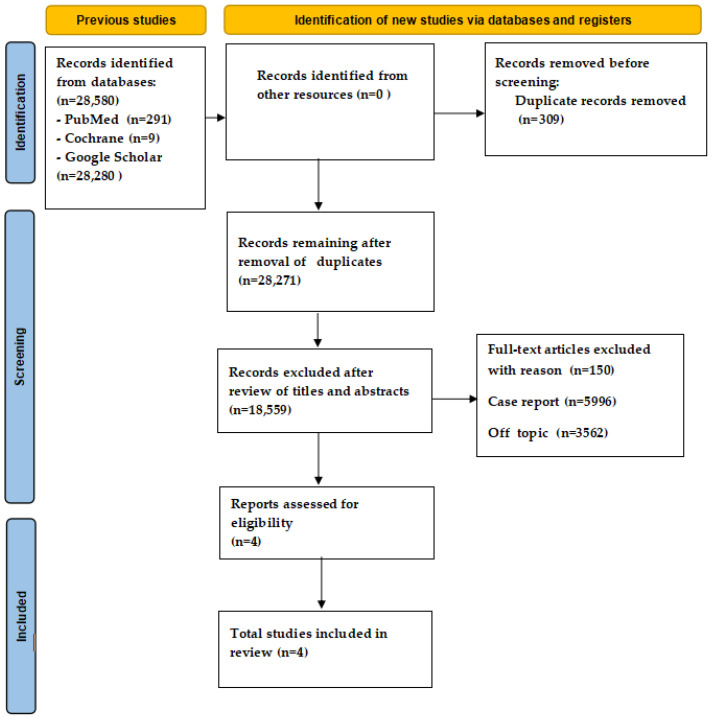
The flowchart diagram for the selection of the studies included in the present analysis.

The search for scientific articles was performed on 16 June 2024, in medical databases such as PubMed, Cochrane and Google Scholar. The data were extracted independently by two reviewers (C.M. and G.M.). A manual search was also performed to improve the pool of articles. The authors discussed the inclusion of each article, and the PRISMA flow diagram shows the flow of information through the different stages of the review process (Figure 1).

The keyword indicators used were “tongue cleaning in children” and “caries and systemic diseases and children”, for which inclusion and exclusion guidelines were drawn up. After preliminary screening and the removal of duplicates, the abstracts and titles of the articles were evaluated to include the articles and their citations that matched the keywords for full-text reading. A total of 154 articles were found to be suitable for full-text reading; of these, 60 focused on the OH protocol for the prevention and treatment of halitosis, 40 related to orthodontic situations, and 50 were not comprehensive about the technique of tongue cleaning used by the study participants. Finally, only four articles were eligible for qualitative analysis and were included in the present review.

### 2.2. Inclusion and Exclusion Criteria

#### 2.2.1. Inclusion Criteria

1. Articles without time limits;
2. Papers in English;
3. Experimental studies on tongue hygiene in children;
4. A minimum number of two decayed, reconstructed or extracted teeth (DMFS/dmfs > 2);
5. Experimental studies with decay-free children who did not practice OH for several days before starting to experiment with different tongue hygiene techniques;
6. Analysis of the bacterial plaque index and salivary *S. mutans* levels at time zero, with re-evaluation a minimum of 7 days after the introduction of tongue cleaning;
7. Experimental studies in humans and in vitro;
8. Reviews and meta-analyses;
9. Randomized controlled trials;
10. Clinical trials.

#### 2.2.2. Exclusion Criteria

1. Case reports;
2. Scientific articles that did not include tongue cleaning in the study protocol;
3. Brief communications;
4. Opinion documents;
5. Inability to access the complete text;
6. Book chapters/proceedings of congresses.

## 3. Results

The search revealed 28,580 total results. Initially, after removing duplicates, the titles and abstracts of the studies related to the keywords were considered to select the articles eligible for the reading of the full text. In total, 28,267 articles were excluded, and only 4 randomized studies, which met the search criteria, were included in the review (Table 1 and Table 2).

Overall, 183 children aged 9 to 12 years (mean age of 10.5 years) were involved in the included studies. The studies by Winnier et al. (2013) [60] and Manju et al. (2015) [61], demonstrated that the total bacterial plaque index and CFU in *S. mutans* saliva were statistically significantly reduced in the OH groups with the brushing and scraping of the tongue at the first checkup after 7/10 days, maintaining or improving this result at the second checkup after 21 days. Winnier et al. divided 45 children into three single-blind experimental study groups; the first group (tongue brushing) and the second group (tongue scraping) had to combine tongue cleaning with OH (teeth and gum brushing) twice a day, while the third group had to brush their teeth without cleaning the tongue. The method of cleaning the tongue by using a scraper and a toothbrush has been shown to be equally effective in reducing the number of cariogenic bacteria; therefore, tooth brushing and tongue scraping were comparable in terms of efficacy in reducing the recorded bacterial plaque index (Silness and Loe) at baseline and after 10 and 21 days [60]. Manju et al., in their single-blind randomized clinical study, divided 45 children into three distinct random groups: a tongue-scraping group, tongue-brushing group, and a group with mouth rinse with prepared saturated saline and nine small tablespoons of table salt in two-thirds of water. All the methods studied in the three groups proved to be effective in reducing the salivary CFU of *S. mutans* compared with the baseline at 7 and 21 days, with no differences among the groups [61]. In the study by Chhaliyil et al., 2020 [62], in three experimental groups, a method for mechanically disintegrating bacterial plaque, easy and simple to employ anywhere, which involves rubbing the gums, teeth and tongue with the index finger and rinsing the oral cable with natural water (GIFTS method) after each meal, snack and intake of acidic or sugary drinks, was compared with conventional OH in the second study group, where the cleaning of the tongue with a toothbrush was always performed in combination with toothpaste (BT method), and with a toothbrush with nano-carbon toothpaste (CT method) in the third study group. The interruption of the organization of recent bacterial plaque, necessary for adhesion and a more complex microbial process, was evaluated as statistically significant. The mechanical action of the index finger, in the test group, at the 10-day checkup, showed that this simple mechanical maneuver was more effective than in the control groups in interfering with the early aggregation of bacterial plaque. In bacterial samples taken from the back of the tongue, the GIFTS and CT hygiene protocols made it possible to particularly lower the number of pioneers of Firmicutes. It was highlighted from the statistical results that the BT cleaning technique achieved the greatest decrease in the means of actinobacteria and phylum proteobacteria. An important abatement in Fusobacterium numbers in the crypts of the tongue microbiota was found, emphasizing that the tested hygiene practices interfere with the process of bacterial aggregation between the purple complex and the red complex. This result was demonstrated by the significant decrease in the phylum Bacteroidetes, especially *Prevotella* and *Porphyromonas*. On the basis of these findings, it could be suggested that OH, in synergy with tongue hygiene, promotes symbiosis in the oral environment [62]. In the study by Sharma et al. (2019) [63], four study groups were compared to evaluate the effects on *S. mutans* salivary colonies of four different types of mouthwash, used after OH twice a day: group 1 performed rinses with 0.2% chlorhexdine gluconate; group 2 performed rinses with xylitol; group 3 performed rinses with antibacterial phytotherapeutic mouthwash (trade name of Himalaya-HiOra); group 4 performed rinses with distilled water. At the first checkup after seven days (without cleaning the tongue), the experimental groups obtained a reduction in *S. mutans* CFU with antibacterial mouthwashes; at the second checkup, at 14 days, with tongue cleaning being performed in all study groups, the introduction of tongue brushing significantly reduced the salivary CFU of *S. mutans* only in the control group (who rinsed the oral cavity with distilled water) [63]. In a previous study, conducted by White et al., the greatest reduction in *S. mutans* CFU was recorded in the tongue-scraping group compared with the saturated saline rinse group, but the groups were adults with permanent dentition [64].

### Risk of Bias

The included works have a low risk of bias; the contribution of new studies will be necessary to confirm the initial endpoint. Table 3 shows the risk of bias of the main articles examined. This review has a relatively low risk of bias.

## 4. Discussion

Among the causes attributable to the onset of caries disease are poor OH, an unhealthy diet and microbiota dysbiosis [65].

An analysis of the literature shows that the most frequent form of oral pathology in children is associated with bacterial plaque and manifests itself as gingivitis and caries [66,67]. The results of this review underline that the greatest reduction in the plaque index and CFU in the saliva of *S. mutans* can be obtained with mechanical hygienic maneuvers, also avoiding or minimizing the use of antibacterial agents, which should be, inter alia, used only if necessary for a short time so as not to alter the balance of the host microbiota, interfere with oral symbiosis and cause drug resistance, especially in children with developing immune systems [68]. Oral diseases and systemic correlations can, therefore, be prevented by combining OH of the tongue at both the domestic and professional levels. In this regard, the mechanical cleaning of the tongue is not always included in the protocols for the primary prevention of caries, while it is an integral part of the OH protocol for the prevention and treatment of bad breath, with conflicting results [69,70,71,72,73,74]. *S. mutans is* released into the saliva from the back of the tongue and dental plaque, and the tongue, as already discussed, carries a higher level of bioburden than the other oral coatings [46,75,76]. In the adult population, to remove more than 42% of oral biofilm (percentage ranging from 24% to 61% depending on the use of manual and electric toothbrushes and the individual’s manual and technical skills) [77,78], it is necessary to combine other OH strategies. The use of toothbrushes and interdental devices is suitable for limiting the FMPS and is becoming the gold standard for the instrumental containment of bacterial plaque, so much so that it is supported by the World Health Organization (WHO) [79,80,81,82]. The inclusion of tongue cleaning, performed regularly during OH, allows for a significant reduction in medium-colonizing bacteria with a substantial reduction in Fusobacterium sp., compromising the maturation of bacterial plaque even in very young children and those without teeth [80,81]. Furthermore, the frequent rinsing of the mouth with plain water after each snack further aids in the removal of food particles and debris from the tongue and teeth and in the destabilization of the oral bacterial biofilm [81]. The frequent cleaning of the tongue and between the teeth is associated with better self-perceived oral health and a reduction in the FMBS. Data from the study by Rickenbacher et al., obtained from a population of 58 children, showed that the introduction of a professional tongue cleaner as an integral part of professional oral prophylaxis was well received by the young patients, and after a month during which they continued to clean their tongue at home, they stated that they wanted to continue in the future. Parents were also sensitized and involved in the project to allow for close collaboration aimed at strengthening preventive intervention [83]. In this regard, involving parents or caregivers in oral screening and the instructions of the complete OH protocol can offer the unique opportunity to educate not only the pediatric population but also the adult one and forge a therapeutic–preventive alliance essential to achieving the goal of large-scale prevention [84,85,86,87,88].

In the Report on the Global State of Oral Health (Global Oral Health Status Report 2023), the WHO requested urgent action to give people with health problems the opportunity to be treated, above all to guarantee prevention, with one ambitious goal of meeting oral health needs globally by 2030. Untreated carious disease of permanent teeth is the most widespread in the world with indecipherable prevalence (approximately 2 billion cases), followed by periodontitis, with about 1 billion people affected, and caries of milk teeth, with about 510 million cases. The prevention of tooth decay and its systemic correlates urgently reflects, therefore, the need in modern society for primary prevention which is easily available, immediate, effective, affordable for everyone and at the same time simple to implement from very early childhood, if not even before birth through oral hygiene and nutritional programs, intervening on modifiable risk factors for the unborn child from “zero” time, i.e., during pregnancy. There is a need for the strengthening of prevention and oral education programs in the first months of life and in pre-school age also through digital means to easily reach new mothers, especially during early childhood, to guarantee currently lacking oral educational projects throughout life [89].

## 5. Tongue-Cleaning Protocols

Below, we propose OH protocols for the mucous membranes and tongue to be practiced with OH for a minimum of two times a day, not neglecting the time before bedtime.

### 5.1. Protocol for Hygiene of Tongue and Oral Cavity in Newborns

In the first months of the baby’s life, it is possible to use the appropriate wipes to clean the oral cavity after each feeding or, alternatively, to use sterile cotton gauze sufficiently soaked in physiological saline water.

To clean a child’s oral cavity, the following steps are recommended:

(1) Sit the child on your lap, facing one side.
(2) Support their head with your arm and one hand.
(3) Use the index finger and thumb of the hand supporting them to open their mouth.
(4) Wrap the gauze around the index finger of the other hand.
(5) Clean the palate, tongue and gums by gently rubbing them, with circular and posterior–anterior movements [62,63].

### 5.2. Tongue-Cleaning Protocol with Toothbrush

After OH with an electric or manual toothbrush, place a tongue brush along the midline of the back of the tongue as far back as possible, avoiding reaching the point that could induce the gag reflex, then slide the toothbrush forward and backward with light pressure, in five sequences, from the back to the tip. Rinse the toothbrush under running water to remove residue, repeat the sequences on both sides of the tongue, and finally rinse the oral cavity vigorously with water to remove any debris and bacteria that have not completely detached [49,60,62]. 

### 5.3. Tongue-Cleaning Protocol with Tongue Scraper

After OH with an electric or manual toothbrush, place the tongue cleaner at the most proximal point to the root of the tongue mantle and perform posterior–anterior movements, gentle but decisive. Repeat the sequence five times, taking care to clean the instrument with water, and finish with a vigorous rinse [49,60,61,62,83].

## 6. Conclusions

Primary prevention is recommended to maintain health and reduce the possibility of disease onset. Daily tongue hygiene, two times a day, during OH, could be considered the gold standard for the prevention of carious disease. Furthermore, this simple mechanical maneuver is assimilated naturally if proposed from birth. The multidisciplinary preventive approach to oral health among professionals, healthcare workers (dental hygienists, pediatricians, nutritionists, etc.) and parents remains essential to protecting and promoting the overall health of the child. This review improves the understanding of the impact of tongue hygiene on controlling the bacteria responsible for the onset of carious disease and its systemic correlates; however, further research with more data is needed to further confirm the findings of this research.

## Figures and Tables

**Table 1:** Articles included in the review.

Authors	Study Typology	Patients Involved	Groups and Method	Checks	Results
Winnier et al., 2013 [60]	Comparative clinical study	45 children, 9–12 years old (divided into 3 random groups)	(1) OH and brushing of tongue; (2) OH and scraping of tongue; (3) OH without cleaning of tongue.	10 and 21 days	The results of the present study show that the tongue-scraping and tongue-brushing groups showed a statistically important low FMPS after 10 days, confirming the result at the 20-day follow-up.
Manju et al., 2015 [61]	Randomized comparative clinical trial	45 children, 9–12 years old (divided into 3 random groups)	(1) Brushing of tongue; (2) clearing of tongue; (3) oral rinses with saturated saline solution.	10 and 21 days	Intragroup comparisons showed a statistically significant reduction in *S. mutans* levels (*p* < 0.01). However, intergroup comparisons did not lead to a statistically significant gap.
Chhaliyil et al., 2020 [62]	Randomized, controlled study	45 children, 10–12 years old (divided into 3 random groups)	(1) Tongue cleansing twice a day with toothbrush and toothpaste (BT); (2) gum and tooth rubbing with index finger tongue cleaning and water swishing (GIFTS); (3) cleansing of oral cavity with finger and nano-carbon toothpaste twice a day (CT).	10 days	GIFTS significantly decreased the salivary number of bacteria compared with BT (*p* < 0.02). Regarding bacterial plaque composition, GIFTS significantly reduced early colonizers, including *Streptococcus*, compared with the BT and CT methods.
Sharma et al., 2019 [63]	Randomized comparative clinical trial	48 children, 9–12 years old (divided into 4 random groups)	Mouthwashes: (1) distilled water; (2) chlorhexidine digluconate; (3) HiOra; (4) xylitol.	7 and 14 days	The inclusion of tongue brushing significantly reduced the salivary *S. mutans* CFU only in the control group (*p* = 0.009) but not in experimental mouthwash groups.

Abbreviations: OH: oral hygiene; *S. mutans*: *Streptococcus mutans*; BT: brushing of the tongue; CT: cleaning of the tongue; CFU: colony-forming unit.

**Table 2:** Change in oral microbiota in children who complete OH by combining tongue cleaning (at least 2 times per day) in the included studies.

	Problem	Intervention/Comparison	Outcomes
Winnier et al., 2013 [60]	Changes in oral microbiota during mixed dentition, with increased susceptibility to caries.	Detection of Silness and Loe plaque index at T0, 10 and 21 days of the study. The plaque index was revealed on specific teeth in the four quadrants: 16, 12, 24, 36, 32, and 44. The deciduous counterparts were analyzed when the permanent teeth were not present in the arch. On each of the four surfaces, a value from 0 to 3 was calculated along the gum line of the tooth. By adding up the data obtained for each tooth, the total, in turn, was divided by all the teeth examined.	The groups that performed tongue scraping and brushing during OH showed statistically significant reductions in the plaque index after 10 days and also after 21 days.
Manju et al., 2015 [61]	Analysis of salivary concentration of *S. mutans* before and after experimenting with tongue hygiene protocols that involved scraping back of the tongue, rubbing tongue with soft toothbrush and vigorous rinsing of mouth with saturated saline solution.	Saliva samples, collected from the subjects before the start, were inoculated in mitis salivarius bacitracin agar and incubated at 37 °C for 48 h. The median *S. mutans* CFU was enumerated. The statistical analysis of the data obtained was performed through the Wilcoxon test, based on ranks, for statistical comparison within each group studied, while the Mann–Whitney U test was adopted for comparison between groups.	There was a gradual decrease in *S. mutans* levels from baseline after the 7th and 21st days. The results showed that all three supplementary OH measures proved effective in reducing *S. mutans* CFU.
Chhaliyil et al., 2020 [62]	Testing of simple tongue-cleaning methods to study their effect on early bacterial biofilm.	At T0 and after ten days, saliva, bacterial plaque and scraped debris samples on the back of the tongue were collected to analyze quantitative polymerase chain reaction (qPCR) and next-generation metagenomic sequencing to compare microbiota taxa between groups.	Simply mechanically disturbing the biofilm without the need for a cleansing agent, every time you eat or snack on food, reduces the early colonizers of the biofilm.
Sharma et al., 2019 [63]	Evaluation of additional effect of tongue brushing and mouth rinsing in addition to regular toothbrushing brushing on saliva CFU of *S. mutans*.	In phase 1, salivary *S. mutans* CFU levels were determined at baseline (after professional oral prophylaxis) and one week after mouthwash use. Phase 2 implemented the same protocol as phase 1 but with the addition of tongue brushing for one week, at the end of which the recording of salivary *S. mutans* CFU levels was performed. The *t*-test was used for intragroup comparison. The intergroup comparison of *S. mutans* CFU was performed by an unpaired *t*-test of the independent sample *t*-test and then ANOVA, followed by the Bonferroni post hoc test.	The inclusion of tongue brushing significantly reduced the salivary CFU of *S. mutans* only in the control group but not in the experimental groups of antibacterial mouthwashes.

Abbreviations: OH: oral hygiene; *S. mutans*: *Streptococcus mutans*; CFU: colony-forming unit; *t*-test: statistical test.

**Table 3:** The risk of bias of single studies. The green symbol indicates a low risk of distortion, while the yellow symbol indicates a moderate risk of distortion.

	Adequate Sequence Generated	Allocation Concealment	Blinding	Incomplete Outcome Data	Registration Outcome Data
Winnier et al., 2013 [60]	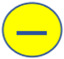	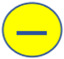	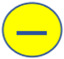	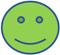	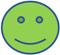
Manju et al., 2015 [61]	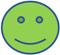	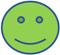	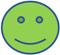	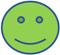	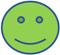
Chhaliyil et al., 2020 [62]	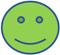	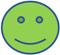	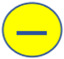	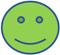	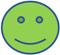
Sharma et al., 2019 [63]	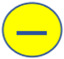	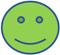	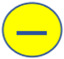	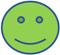	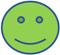
